# Groove Loss Time: A Novel Wound Leakage Test for Sutureless Clear Corneal Cataract Wound Incision

**DOI:** 10.3390/jcm14124091

**Published:** 2025-06-10

**Authors:** Sunjin Hwang, Moonsu Kim, Jooyoung Yoon, Eun Hee Hong, Yong Un Shin, Min Ho Kang

**Affiliations:** Department of Ophthalmology, Hanyang University Guri Hospital, Hanyang University College of Medicine, Guri 11923, Republic of Korea; sunjin1989@hanyang.ac.kr (S.H.); kmoon0902@naver.com (M.K.); cec1204@naver.com (J.Y.); ehhong@hanyang.ac.kr (E.H.H.); yushin@hanyang.ac.kr (Y.U.S.)

**Keywords:** clear corneal incisions, wound hydration, groove loss time

## Abstract

**Background:** This study introduces a novel quantitative method—groove loss time (GLT)—to objectively assess wound leakage following cataract surgery. **Methods:** In this prospective, single-center study, 70 eyes of 70 patients undergoing cataract surgery via CCI were enrolled. Wound sealing was evaluated by measuring the GLT, defined as the duration the stromal groove remains visible after corneal hydration. GLT was categorized into five grades: ‘water-tight’ (>10 s), ‘excellent’ (>5 s), ‘good’ (3–5 s), ‘bad’ (1–2 s), and ‘poor’ (<1 s). Intraocular pressure (IOP) was recorded at four time points: preoperatively, immediately post-surgery, 3–4 h postoperatively, and on postoperative day one. In select cases, anterior segment optical coherence tomography (AS-OCT) was used to confirm wound architecture. **Results:** All patients demonstrated a GLT longer than 5 s, corresponding to water-tight or excellent wound sealing. Mean IOP values were 16.08 ± 3.61 mmHg preoperatively, 29.48 ± 11.13 mmHg immediately post-surgery, 16.38 ± 5.45 mmHg at 3–4 h, and 16.65 ± 4.36 mmHg on the day after surgery. No cases of postoperative endophthalmitis, anterior chamber loss, or hypotony were observed. **Conclusions:** The GLT method provides a simple, objective, and effective tool for evaluating wound integrity in CCIs, ensuring optimal sealing and enhancing postoperative safety.

## 1. Introduction

In modern cataract surgery, among the various methods used to create incisions, the clear corneal incision (CCI) is the most common. The benefits of CCI include shorter surgery duration, better maneuverability of surgical instruments, reduced cost of surgery without sutures, decreased induction of corneal astigmatism from a suture, and reduced risk of unexpected bleeding [1]. There are several methods to close the incision, including interrupted sutures, corneal hydration, and tissue adhesives [2]. Among these, closure through corneal hydration is widely used [3]. However, this method may not completely seal the incision [4], potentially increasing the risk of hypotony or endophthalmitis [3,5,6]. Therefore, it is crucial to verify the self-sealing of the incision at the end of surgery.

Typically, the incision site is assessed by applying pressure with a Weck cell sponge; however, this method has the limitation of inconsistent force, hindering reliable evaluation of the seal. In CCIs, a groove along the wound can be observed under a surgical microscope due to epithelial defects on the anterior surface, separation of Bowman’s layer, and part of the anterior stroma. If wound leakage is present, the leaked aqueous humor fills the groove, rendering it invisible under the microscope. The intraocular pressure (IOP) required to maintain a sealed state varies depending on the length and shape of the CCI [7,8]. Wound leakage usually occurs when the IOP falls below the pressure required to maintain a sealed state. Self-sealing of a CCI by stromal hydration functions like a check valve at the level of Descemet’s membrane and the posterior stroma.

Therefore, we propose a method to measure the time during which the wound groove remains visible, allowing a quick and simple assessment of the self-sealing status of the CCI, while also providing an objective way to describe the degree of sealing.

## 2. Materials and Methods

### 2.1. Study Design

This was a prospective, single-center study approved by the Institutional Review Board of Hanyang University Guri Hospital (IRB number: 2025-01-005-001, approved on 28 January 2025). The study was conducted after thoroughly explaining the details to the patients and obtaining their written informed consent. All procedures adhered strictly to the ethical guidelines outlined in the Declaration of Helsinki.

### 2.2. Participant Characteristics

The study enrolled patients who underwent phacoemulsification and intraocular lens (IOL) implantation using the CCI technique for cataract at Hanyang University Guri Hospital between 1 February 2025 and 31 March 2025. Consecutive unrelated patients in need of cataract extraction were enrolled in the study. Each patient underwent a comprehensive ophthalmologic evaluation, which included an assessment of best corrected visual acuity (BCVA), IOP measurement, examination of the crystalline lens, dilated fundus evaluation, and biometry for IOL calculations. In some patients, anterior segment Optical Coherence Tomography (AS-OCT) was used to confirm proper wound sealing. Patients with primary ocular hypertension or glaucoma, which could affect IOP measurements, and those with corneal diseases that could impact the observation of wound leakage were excluded. Additionally, patients with systemic conditions, such as uncontrolled diabetes, which could influence wound healing, were also excluded from the study. All patients were followed for six months to monitor for postoperative endophthalmitis.

### 2.3. Surgical Techniques

Phacoemulsification was performed by an experienced anterior segment surgeon (M.H.K.) using a Centurion System (Alcon Laboratories, Inc., Fort Worth, TX, USA). All cases were performed under topical anesthesia with 0.5% proparacaine. A two-plane, square-shaped CCI was placed temporally with a 2.8 mm double-blade corneal knife. An additional puncture for the second instrument was made at the 6 or 12 o’clock position of the limbus based on the laterality of the eye. After injecting viscoelastic solution (1% Hyalu injection, Hanmi Pharmaceutical Co., Ltd., Seoul, Republic of Korea) through the side port, a 5.5 mm continuous curvilinear capsulorhexis was created using a 25-gauge needle. Hydrodissection and hydrodelineation were carried out with a balanced salt solution (BSS). Phacoemulsification was performed using the phaco-chop technique, and irrigation/aspiration was completed through the main wound. Then, viscoelastic material was injected into the anterior chamber, with a single-piece monofocal IOL (ZCB00, Johnson & Johnson Vision, Santa Ana, CA, USA) injected using the cartridge. After completely removing the viscoelastic material from the anterior and posterior chambers, the anterior chamber was filled with BSS, and corneal hydration was used to attempt closure of the incision (both main and side port incisions). After surgery, all patients were given topical antibiotics to be applied four times daily for two weeks. Topical steroids were administered 4–8 times per day, with the frequency gradually decreased over time. Additionally, topical nonsteroidal anti-inflammatory drugs were prescribed twice daily for two months postoperatively.

### 2.4. IOP Measurement

IOP was measured using a Tonopen (Reichert Technologies, Depew, NY, USA). A single examiner (M.H.K.) performed two measurements for each time point, and the average of the two values was used for analysis. IOP was measured at four time points: before surgery, immediately after surgery just before removing the speculum, 3–4 h postoperatively, and on the day after surgery. During each measurement, the upper eyelid was carefully lifted by pinching it with fingers to avoid any pressure on the eye.

### 2.5. Groove Loss Time

**Figure 1:**
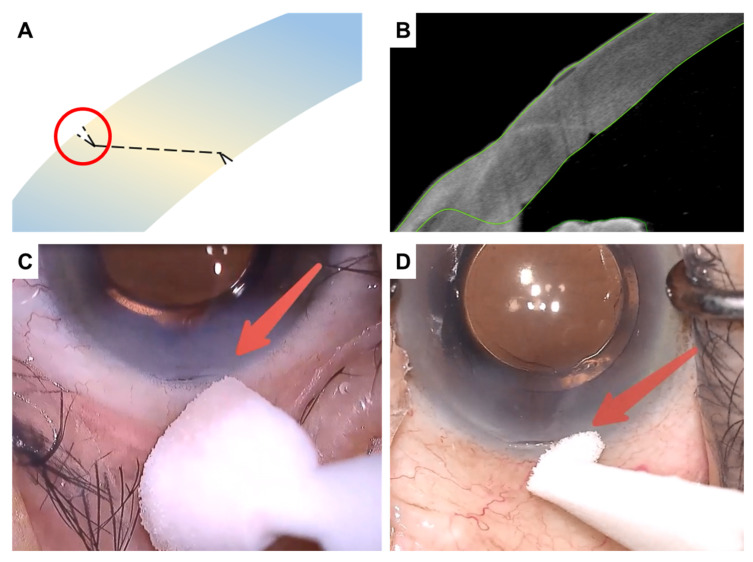
(**A**) Schematic representation of the groove formed after CCI (clear corneal incision) wound sealing. The groove is highlighted by the red circle. (**B**) Anterior segment optical coherence tomography confirms proper wound healing. (**C**) Fluid leakage from the wound fills the groove, causing it to gradually disappear. We define the groove loss time as the interval from the initial appearance of the groove after drying until the groove completely disappears due to fluid leakage. (**D**) The groove can be observed as the wound dries.

Epithelial defects, along with small dehiscence in Bowman’s layer and the anterior stroma, are often seen in CCIs. As a result, when the wound is dry, a groove can be observed along the wound site under a microscope. Depending on the degree of wound leakage, this groove will gradually disappear after the wound is dry. The time taken for the groove to disappear is referred to as the ‘groove loss time’, and this parameter allows assessment of the degree of wound leakage (Figure 1).

We classified wound sealing into five grades as shown in Table 1: ‘water-tight’ when the wound groove is visible for more than 10 s, ‘excellent’ when visible for more than 5 s, ‘good’ for 3 to 5 s, ‘bad’ for 1 to 2 s, and ‘poor’ when the groove is not observed or disappears within 1 s. Before measurement of the groove loss time, the IOP should be approximated using gentle pressure applied to the upper eyelid with a sterile cotton swab, avoiding direct contact with the globe. When the IOP is judged to be higher than normal, moisture from one end of the wound groove should be removed using a Weck cell sponge. There is no need to press on the wound or wipe the entire wound. After the Weck cell sponge is removed from the incision, the time the groove remains visible is measured.

If the groove disappears in less than 3 s, time can be saved by releasing some aqueous humor to lower the IOP and re-evaluate. If the groove is maintained for more than 5 s at higher-than-normal IOP, the incision is judged to be self-sealing. We considered the incision sufficiently closed when it was rated as either ‘water-tight’ or ‘excellent’ by this method. In most cases, the IOP immediately after surgery was measured between 20 and 30 mmHg, with an average of 21 mmHg using a Tonopen (Reichert Technologies, Depew, NY, USA).

### 2.6. AS-OCT Measurement

Among patients, a small portion who provided certain informed consent underwent AS-OCT imaging with Anterion (Heidelberg Engineering GmbH, Heidelberg, Germany) of the operated eye in the ophthalmology outpatient clinic immediately following surgery. The AS-OCT findings were classified into five categories: completely intact wound integrity, endothelial and epithelial gaping, loss of wound coaptation, localized detachment of Descemet’s membrane, and endothelial misalignment.

### 2.7. Statistical Analysis

Data analysis was performed using SPSS software, version 29.0 (IBM Corp., Armonk, NY, USA). All values are presented as mean ± standard deviation. IOP changes at each time point were analyzed using one-way ANOVA, and pairwise comparisons between time points were conducted using paired *t*-tests. A *p*-value less than 0.05 was considered significant.

## 3. Results

### 3.1. Baseline Characteristics

A total of 55 eyes of 55 patients were enrolled in this study. All patients underwent phacoemulsification with IOL implantation in the bag, and there were no posterior capsule ruptures or radial tears. Among the 55 patients, the average age was 68.11 years with 30 males and 21 right eyes. LogMAR BCVA improved significantly from 0.71 ± 0.57 before surgery to 0.19 ± 0.36 after surgery. The preoperative nucleosclerosis grade averaged 3.32 ± 0.52, and the operation duration was 25.40 ± 9.74 min. Preoperative central corneal thickness was measured at 535.24 ± 47.57 μm, and the axial length was 23.76 ± 0.99 mm (Table 2). There were no cases of postoperative endophthalmitis, anterior chamber loss, or hypotony.

### 3.2. IOP Measurements

**Figure 2:**
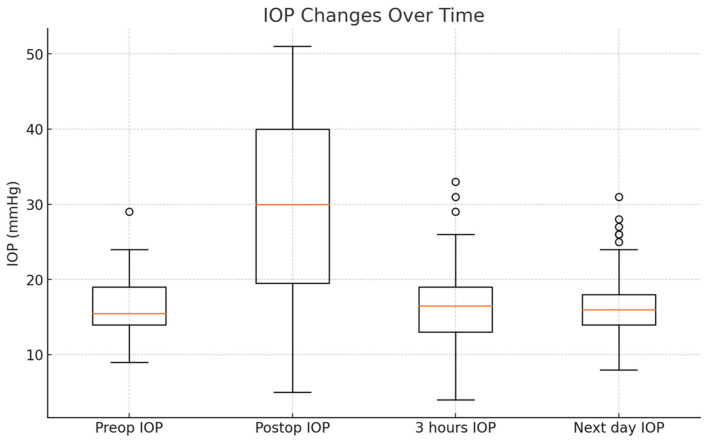
A box-plot illustrating the changes in IOP over time before and after surgery. The red line inside each box represents the median IOP, the top and bottom edges of each box correspond to the third and first quartiles, and the whiskers extend to the minimum and maximum values (within 1.5 times the interquartile range, with any outliers beyond that shown as individual points if applicable).

The preoperative mean IOP of the 70 patients who underwent cataract surgery was 16.08 ± 3.61 mmHg. One patient had an elevated IOP of 29 mmHg, but surgery proceeded as there were no specific issues. Immediately after the surgery, at the point when the IOP was expected to be in the higher-normal range upon palpation, the groove loss time was measured, and the IOP measured by Tonopen at that time was approximately 29.48 ± 11.13 mmHg. Although one patient had an IOP as high as 50 mmHg right after surgery, the pressure had returned to the normal range at 16.38 ± 5.45 mmHg at discharge. Only one patient had an IOP higher than 30 mmHg at discharge, but IOP-lowering drops the next day resulted in a pressure within the normal range. Three cases had IOPs lower than 6 mmHg at discharge, but all showed normal IOP the next day. When the IOP was measured with Tonopen the day after surgery, the average IOP was about 16.65 ± 4.36 mmHg, with no cases of hypotony (Table 3, Figure 2).

### 3.3. AS-OCT Interpretation

**Figure 3:**
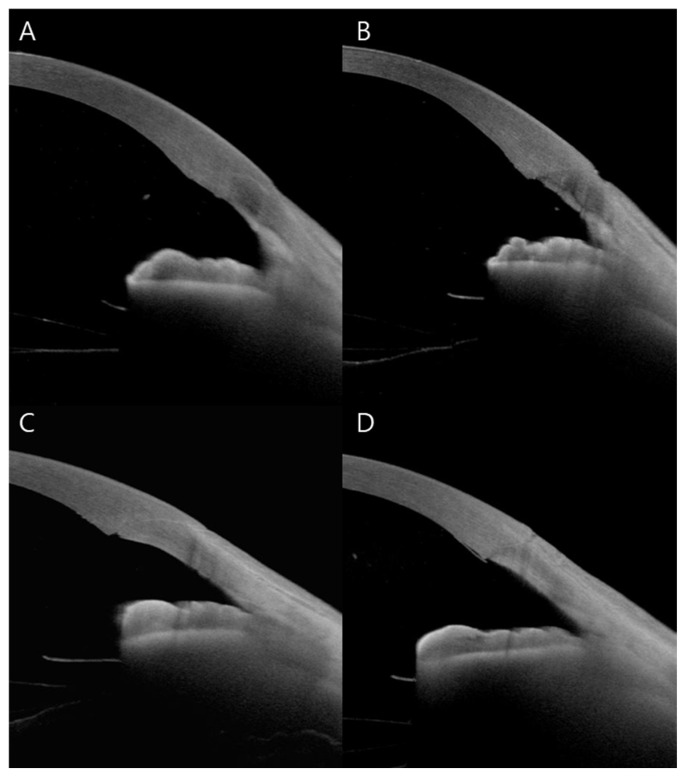
AS-OCT images of CCIs immediately after surgery. All incisions were created using a two-plane technique. Representative images illustrate different wound healing states. (**A**) completely intact wound integrity, (**B**) endothelial misalignment with epithelial gaping, (**C**) endothelial gaping, (**D**) local detachment of Descemet’s membrane accompanied by epithelial gaping.

A total of 16 eyes of 16 patients underwent AS-OCT. Among these, four eyes (25%) demonstrated completely intact wound integrity. Epithelial gaping was observed in eight eyes (50%), endothelial gaping in five eyes (31%), local detachment of Descemet’s membrane in three eyes (19%), and endothelial misalignment in three eyes (19%). There were no cases of loss of wound coaptation (Figure 3).

## 4. Discussion

This study demonstrates that GLT can serve as an objective indicator of wound integrity in CCIs during cataract surgery. By timing how long the hydrated stromal groove remains visible, the surgeon can identify the point of optimal wound sealing before concluding the case. In our series, when GLT was maintained for more than 5 s (classified as ‘water-tight’ or ‘excellent’ seal), postoperative IOP remained stable and no wound-related complications such as endophthalmitis, anterior chamber shallowing, or significant hypotony were observed. This suggests that achieving a GLT beyond a critical threshold (5 s in this study) correlates with a well-sealed incision and favorable postoperative stability.

Performing corneal hydration after CCI shortens surgical duration, is cost-effective, and eliminates the need for suture removal, making it highly convenient. However, the procedure has a risk of postoperative endophthalmitis [9,10]. Therefore, it is important to confirm proper wound sealing immediately after surgery through wound hydration. A study has shown that stromal hydration significantly reduces fluid ingress into the anterior chamber compared to cases without stromal hydration, highlighting its key role in wound closure [11]. However, Calladine et al. [1] observed through anterior segment OCT that CCI wound architecture integrity often deteriorates at low IOP levels, with findings such as endothelial and epithelial gaping, loss of coaptation, local detachment of Descemet’s membrane, and endothelial misalignment, which hinder wound apposition. In practical terms, if the eye is hypotonic at the end of surgery, the incision may gape open despite hydration. This reinforces the importance of achieving a sufficiently firm globe (we adjusted IOP to a higher-normal level by palpation using a cotton swab before measuring GLT) when testing the wound. In our protocol, after hydration, we ensured the eye was palpably firm (around 20–30 mmHg, confirmed by Tonopen) before timing the groove’s persistence. This simulates normal physiologic IOP and even a slight Valsalva or eye squeeze in the early postoperative period. If the wound holds at that pressure (GLT > 5 s), it implies that minor fluctuations in IOP (for example, when the patient coughs, moves, or rubs the eye) are unlikely to cause it to leak. Indeed, no leakage was noted in any eye with GLT ≥ 5 s in our series, and only transient hypotony occurred in a few cases immediately after surgery—all of which resolved by the next day as the wounds self-sealed. Moreover, our AS-OCT findings demonstrated that the overall wound architecture was relatively well preserved immediately after surgery. Although certain wound abnormalities such as endothelial misalignment and local detachment of Descemet’s membrane were observed, the prevalence was approximately one-third lower compared to previous reports [1], which documented these findings in up to 60% of cases. While the sample size in our study was limited, the data suggest a relatively favorable early postoperative wound configuration.

Prior studies comparing scleral tunnel incisions (STIs) to CCIs provide further context. STIs are thought to be more stable due to the support of overlying sclera and conjunctiva, and generally show higher immediate postoperative IOP [8,9]. Hayashi et al. [8] reported that eyes with scleral tunnel incisions had higher IOP in the first hour after surgery than eyes with CCIs. By 30 min post-op, mean IOP in the CCI group had fallen below the preoperative baseline in their study. Importantly, they observed hypotony (<10 mmHg) in about 10.9% of eyes after CCI cataract, whereas no eyes in the STI group experienced hypotony. We see a parallel in our results: a few CCI eyes had low pressure shortly after surgery, but with a well-formed incision and GLT above 5 s, the wounds sealed themselves rapidly, and IOP returned to normal by the next follow-up. In fact, only one eye in our series had what we classified as postoperative hypotony despite a GLT > 5 s, and that eye’s IOP normalized by the next day without intervention. This raises the possibility that by targeting a certain GLT threshold (e.g., ≥5 s), surgeons could achieve wound closure integrity on par with that of STIs, thereby combining the speed and convenience of CCI with the stability traditionally associated with STI.

The architecture of the corneal incision can also influence wound leakage. Although there have been numerous reports suggesting that three-plane square incisions can reduce wound leakage [12,13], a perfectly square three-plane incision is challenging. In practice, only about 30% of attempts to create a precise three-plane incision are successful. Moreover, even well-constructed triplanar beveled wounds may not fully resist leakage when subjected to external pressure or fluctuating IOP [13]. The size of the incision also impacts wound leakage. Incisions that are too small can become unstable under stretching forces, leading to larger, more vulnerable wounds prone to leakage [2,14]. Additionally, smaller incisions are not inherently resistant to leakage, which can occur at the side port [15]. In the present study, we performed cataract surgery using a 2.8 mm two-plane square CCI and confirmed the absence of wound leakage by assessing groove loss time.

Suturing can also be employed to close CCI wounds. However, whether sutured incisions provide better wound sealing than stromal hydration remains unclear [2]. One study has shown that up to 30% of sutured incisions exhibit wound leakage [6], and approximately two-thirds of cases involving sutured two-plane incisions demonstrated wound site disruption or weakening [16]. Suturing is time-consuming and may lead to complications such as subconjunctival hemorrhage, foreign body sensation, and eye irritation [2,6]. Additionally, loosened or broken sutures can increase the risk of leakage or infection [17,18,19]. Tight sutures can cause wound distortion, potentially inducing astigmatism and decreasing visual acuity [20]. If sutures are left in place too long, corneal epithelialization may occur [21], and one study has reported corneal infection in two-thirds of cases even after removing broken sutures [18]. Given the variability in outcomes and potential complications associated with suturing, CCIs may offer more advantages. Therefore, assessing proper wound sealing through groove loss time could serve as a more effective method for ensuring optimal wound closure.

In clinical settings, incorporating GLT as a standard method to assess wound integrity seems practical, as it requires no specialized equipment beyond the standard tools used for stromal hydration. Measuring the duration of the visible hydration groove in the corneal stroma can be easily done by the surgeon or an assistant using a stopwatch, or even by counting seconds manually. To ensure consistency, a single observer conducted the timing for all cases in our study. We identified a practical threshold of 5 s; all eyes where the hydration groove persisted for 5 s or more experienced uncomplicated postoperative courses, maintaining stable IOP without evidence of wound leakage or infection. Thus, GLT can serve as a straightforward indicator: if the groove disappears quickly, implying fluid leakage, the wound requires further sealing, whereas a persistent groove suggests adequate closure. This provides a quantitative measure to complement the traditional qualitative judgment of wound integrity, offering reassurance in busy surgical environments.

However, there are some limitations. First, timing can be somewhat subjective, particularly in borderline cases, potentially introducing variability among observers. Additionally, the accuracy of GLT depends on the hydration technique and IOP at measurements. While we standardized the pressure through palpation using a cotton swab, real-world variability in pressure could affect GLT results; lower IOP may falsely prolong groove visibility, while excessively high IOP might falsely shorten it. Thus, maintaining consistent IOP during the test is crucial. Before measuring GLT, we first release a small amount of aqueous humor to lower any abnormally high IOP into the normal range. This prevents an unnaturally short GLT caused by excessive IOP. However, fluid leakage depends on more than IOP alone—incision shape, remaining tissue hydration, local inflammation, individual healing response, and surgical technique all affect how quickly fluid egresses. Therefore, while GLT gives a reproducible, objective measure of how well the wound seals under controlled pressure, it should not be used in isolation. A full evaluation of wound integrity must include these other factors, and future work should examine how they influence both GLT and clinical outcomes. Additionally, our study did not directly compare GLT to other objective methods like the intraocular dye leakage test, ultrasound biomicroscopy, or Seidel tests. Confirmation through such modalities could strengthen the validity of GLT. Moreover, the thresholds we proposed were determined empirically, and further refinement through larger-scale studies considering varying incision sizes or configurations might be necessary. Finally, while no adverse effects arose from GLT measurement in our study, excessive hydration in an attempt to achieve longer GLT durations might cause tissue edema or complications such as Descemet’s membrane detachment. Therefore, GLT should guide hydration appropriately rather than encourage excessive measures. In addition, while we performed stromal hydration at both the main incision and the side port, our integrity assessment was applied only to the main CCI wound. We did not specifically evaluate side port sealing, which could theoretically contribute to postoperative leakage or hypotony. Future studies should include formal side port integrity checks to confirm that all incision sites remain water-tight.

In conclusion, GLT represents a simple, effective, and objective way to verify wound integrity after CCI. A sustained hydration groove signals sufficient wound closure to maintain stable postoperative IOP and prevent postoperative endophthalmitis. Compared to traditional methods like the Seidel test, GLT provides a standardized and sensitive way to confirm wound tightness. Our results indicate that GLT can enhance surgical confidence, potentially reducing complications such as hypotony or infection. Although GLT should complement rather than replace proper wound construction techniques, it could become a valuable part of standard cataract surgery protocols pending further validation from larger, multicenter trials integrating advanced imaging modalities.

## Figures and Tables

**Table 1:** Classification of the degree of self-sealing in clear corneal incisions based on the time during which the wound groove is not observed (groove loss time).

Degree of Wound Self-Sealing	Groove Loss Time
Water-tight	>10 s
Excellent	>5 s
Good	3~5 s
Bad	1~2 s
Poor	<1 s

**Table 2:** Baseline demographics of enrolled patients.

	Total (*N* = 70)
Age (years)	67.45 ± 10.59
Sex (male)	37
Laterality (right)	32
LogMAR BCVA (preoperative)	0.66 ± 0.58
LogMAR BCVA (postoperative)	0.17 ± 0.33
MRSE (D)	−0.51 ± 2.17
Nuclear firmness	3.31 ± 0.65
Surgery duration (min)	23.71 ± 9.35
CCT (μm)	535.61 ± 46.51
Axial length (mm)	23.96 ± 1.18

BCVA = best corrected visual acuity; CCT = central corneal thickness; D = diopter; LogMAR = logarithm of minimal angle of resolution; MRSE = manifest refraction spherical equivalent.

**Table 3:** The changes in IOP over time before and after the cataract surgery.

	Preoperative	Immediate Postoperative	3 h After Surgery	1 Day After Surgery	*p*-Value
IOP (mmHg)	16.08 ± 3.61	29.48 ± 11.13	16.38 ± 5.45	16.65 ± 4.36	<0.001

## Data Availability

The data presented in this study are available on reasonable request from the corresponding author. The data are not publicly available due to patient privacy and ethical restrictions.
